# Primary Health Centres: Preferred Option for Birthing Care in Tamilnadu, India, from Users’ Perspectives

**Published:** 2015-03

**Authors:** T.P. Jayanthi, Saradha Suresh, P. Padmanaban

**Affiliations:** ^1^Department of Community Medicine, Government Kilpauk Medical College, Tamilnadu, India; ^2^Institute of Child Health, Tamilnadu, India; ^3^National Health System Resource Centre, Ministry of Health and Family Welfare, Government of India, New Delhi, India

**Keywords:** Birthing care, Institutional deliveries, Primary health centres, Quality of care, India

## Abstract

Tamilnadu state of India witnessed an increasing trend of institutional deliveries since the beginning of 1990s, with decline of domiciliary deliveries to nearly zero now. Among the institutional deliveries, a shift has been observed since 2006 wherein primary health centres (PHC) have shown a four-fold increase in the number of deliveries while other public and private health facilities showed a decline, despite equal access by people to all categories of health facilities. A qualitative study was designed to explore the determinants that led to increased preference of PHCs for birthing care. In-depth interviews and FGDs were conducted with recently-delivering women and their spouses. User-friendly ambience, courteous attitude and behaviour of staff, good infrastructure, availability of qualified staff, and relative absence of informal payments have contributed to increased preference for birthing care in PHCs. Barriers to seeking care from secondary and tertiary-level public hospitals and private hospitals have also made women prefer PHCs.

## INTRODUCTION

Access to safe motherhood care is the right of every woman, and it is the responsibility of the welfare state to provide quality maternal health services. Despite a 59% decline in maternal deaths during the last decade, India contributes to 19% of the global maternal deaths (1). The Government of India has started addressing the issues on maternal and child-care through national programmes as early as 1952. Since 2005, the Government of India, through its National Rural Health Mission, provides technical and funding support to all states in the country for infrastructural strengthening of health facilities, deploying human resources, upgrading their skills, and streamlining drug logistics and supplies. One of the key strategies of the mission to reducing the maternal mortality ratio (MMR) is to promote institutional deliveries to ensure that women have safe birthing care (2).

Tamilnadu is one of the socially- and economically-progressive states in India, with a population of 72.12 million, of which 44% lives in urban area, with a female literacy rate of 73.86% (3). The state is divided into 32 Revenue Districts, of which Chennai is entirely urban, and the rest of the districts are of a rural-urban mix (4). The State fairs well in terms of indicators relating to maternal and child health. The State's population policy in 1993, which recommended a goal of 90% institutional deliveries by 2000, paved the way to increase in institutional deliveries from 56.84% in 1991 to 98% in 2012 (4,5). Tamilnadu stands second, only next to Kerala, its neighbouring state which has achieved 100% institutional deliveries (6).

Maternal healthcare services in Tamilnadu are provided both by public and private sector. The public sector provides services through the Health Subcentres (HSCs) and PHCs in the rural areas and the urban health centres in the urban areas at the primary level. District and subdistrict hospitals provide care at the secondary-level and teaching institutions and their attached hospitals at the tertiary level (7). Secondary and tertiary-level hospitals function as referral hospitals. Women have direct access to all levels of health facilities for birthing care. Services are provided free of charge in the public facilities. The private facilities range from small nursing homes, corporate hospitals to NGO-run hospitals and private medical college hospitals, which charge user-fees.

Prior to 2006, around 43% of the deliveries were conducted in the private institutions, 42% in the secondary and tertiary-level public hospitals, 7% each in the PHCs and HSCs, and a little above 5% had domiciliary deliveries. An analysis of institutional deliveries by sector from 2006 onwards showed a different picture. While HSC and domiciliary deliveries declined to less than 1%, a four-fold increase was observed in PHCs, marginal decline in secondary and tertiary hospitals and, surprisingly, deliveries in the private sector declined by 10 points (4,5,8).

What led to this changing trend in choice of facilities for birthing care and the reasons for preferring the PHCs?

Historically, Tamilnadu is well-known for its innovations in the field of maternal and child health. Very little information is available on women's perception of quality and satisfaction and their choice of facility for birthing care. Such information is essential for evaluating the maternal health services.

This study was designed to explore women's perceptions of quality, their satisfaction, and reasons for preferring PHCs for birthing care over other facilities.

## MATERIALS AND METHODS

A qualitative study design was chosen. Individual in-depth interviews (IDIs) were conducted with women and their spouses as it was considered the ideal technique which would provide a deeper understanding into how they perceived birthing care facilities and factors influencing their perceptions. One focus group discussion (FGD) per study district was conducted to understand group and community norms and also for triangulating information with more than one type of data-collection technique.

Three districts in Tamilnadu were chosen—one with a high MMR (>120), the other with a low MMR (<60), and the third with an average MMR (=60). Catchment area of one PHC area (around 30,000 people) was chosen from each district as the study area. This method of selection was adopted as MMR would serve as a proxy indicator for quality of birthing care. List of women who had delivered two to three months back, along with their place of delivery, was obtained from the village health nurses (VHN). Two women who had delivered in each category of public facilities and private hospitals were identified for IDI from each of the study areas. Care was taken to include women who had one, two, or more children. Interviews were also conducted with spouses of women who had delivered 2-3 months back (n=6).

Total number of women interviewed was 24 distributed as follows:

- PHCs (n=6)
- District and subdistrict hospitals (n=6)
- Teaching institutions (n=6)
- Private hospitals (n=6)

### Focus group discussion

One FGD was conducted in each of the study areas with 8 to 10 women who had delivered two to three months back. It was ensured that there was equal representation of women who delivered at all levels in the public sector and in the private sector. Women in the age-group of 20 to 35 years were selected and had representation from all levels of socioeconomic status.

IDIs and FGDs were conducted in common places, convenient to the participants, ensuring privacy and confidentiality. The study was conducted between May 2012 and September 2012. A guide was developed to ensure consistency, with key focus on choice of facility for birthing care, perceived quality of care, and out-of-pocket expenses incurred. Probes were built into the guide to allow for a thorough understanding of the topic.

All IDIs and FGDs were conducted by the first author and audio-recorded. She was assisted by a note-taker who recorded non-verbal behaviour. Audio-recorded IDIs and FGDs were transcribed and translated into English. A framework analytical approach was used for data analysis. This process involved a number of interconnected stages beginning with: familiarization with data; identifying a thematic framework; indexing and sorting quotes and placing them under the appropriate thematic category, mapping, and final interpretation. This analysis was done manually. Initially about 5 IDIs and 1 FGD were coded inductively, following which a coding scheme was developed. The remaining IDIs and FGDs were coded using this coding scheme. Segments of text that were related to a common theme were pieced together and, in this manner, emergent themes were identified. Finally, quotes were selected from transcripts that best helped illustrate the themes being described.

### Ethics

Permission to conduct the study was obtained from the Government of Tamilnadu, and ethical clearance was obtained from the Institutional Review Board of the Institute of Child Health, Chennai. Informed written consent was obtained from all study participants.

## RESULTS

Information shared by women and their spouses were based on their personal experiences during the recent and previous pregnancies. Five major themes emerged which best explained the reasons behind choosing facilities for birthing care (Box).

### Birth preparedness

#### Choice of health facility

Planning for delivery starts right from the antenatal (AN) period itself. Women understand that deliveries should be conducted by a skilled person in an institution. A clean facility with staff members who were friendly and responsive to the needs of the women was considered an ideal facility.

Choice of the facility depends on various factors, like accessibility, perceived quality of care, facilities available, and the individual's financial status. The place to deliver is mostly decided by the woman but has to be accepted by the family members. Women's personal experience during AN check-ups and the opinion they get about the birthing services from their friends and relatives help decide on the choice of facility for birthing.

The village health nurses (VHN) who are the first point of contact in the care continuum had a role in motivating women to deliver in the PHCs. Many women said, “VHN provides AN check-up, gives medicines, she tells us what we should eat, refers us for scan and to come to the PHC for delivery.”

Culturally, women go to their mother's place for their first delivery, and the expenses are borne by her parents. Here, the choice of the facility is made by the mother of the pregnant woman. In a few instances, they chose private sector as they perceive that the quality is good and also to satisfy their in-laws who would accept that good care has been provided. In spite of such cultural practices, many women had convinced their families and delivered in the PHCs because they felt that the care provided in the PHC is as good as in the private facilities.

Women who participated in the FGDs in all the three districts uniformly stated that PHCs were the preferred option for delivery. In most instances, women accessed public referral hospitals and private hospitals only when PHCs referred them for complications. Very few women planned to deliver in the secondary and tertiary hospitals because they are equipped to mange complications. Private sector was chosen by a few women for perceived good care.

**Box. Major themes explaining various reasons for choosing birthing care facilities**

**Table:** 

Theme	Subtheme
Birth preparedness	Decision-making and choice of health facility Complication readiness
Perceptions on quality of care	Courteous attitude and interpersonal behaviour of staff Emotional support Cleanliness of health facility Prompt care and confidence in treatment
Familiarity and proximity to their residence	Familiar to the staff and facility
Infrastructure and service provision	Physical infrastructure Human resources Medicines, supplies, and services Maternity benefit funds
Cost of care	Hospital expenses Other expenses Informal payment

#### Complication readiness

As a part of birth preparedness, women safely kept their AN card, investigation reports, and kept some clothes ready for use. Most of them did not keep money ready since they had planned to deliver in the PHC where money is not required.

At times of complications, PHCs referred women to referral hospitals in the public sector, and families were in a dilemma where to go. Thinking about the complications and their concern about the quality of care, some opted for private hospitals even if they did not have money while others went to the referred centres. In both circumstances, they took much time and mobilized funds by borrowing money or by pledging jewels at high rate of interest to meet the expenses in seeking care.

### Women's perceived quality of care

#### Courteous attitude and interpersonal behaviour of staff

Courteous and empathetic attitude of the staff emerged as the key determinant for women choosing a facility for birthing care. A uniform statement was made about PHCs by all the mothers “the staff members were very friendly, they understand our problems, they answer to all our questions.” It was not about one category of staff but the entire team, right from the doctors to the cleaning staff.

A good rapport existed between the staff and family members of the women delivering in the PHCs. Even spouses waiting outside the labour room in the hospital premises were informed about the progress of labour. The cordial relationship between the families and the PHC staff was quoted as an important reason for choosing the PHC for subsequent delivery. The emotional support provided by the staff nurses and ANMs at PHC was appreciated.

During the interview, one of the spouses said, “hospitals in general are scary for the public. I felt ease as soon as I came into the PHC because of the kind attitude of the staff. I felt safe to leave my wife under their care.”

A woman who had delivered in the PHC said, “I have come here for both of my deliveries, never they have been angry with me or scolded me—staff members are very kind and friendly, far better than GH (tertiary centres).”

Another woman said, “My previous delivery was in a medical college hospital, now I delivered in the PHC; there the care was not good, and they will not respond to our questions…. Here, it is nice, doctors and nurses talk to us nicely.”

While sharing experiences about the referral hospitals, women felt that it was very difficult to access the services since no one was available/willing to guide them. One of the mothers who had delivered in a tertiary centre said, “If we ask them anything …they start shouting at us or say “go and sit”, it's a torture to be there.”

The attitude of staff in the private hospitals was considered to be good. No special appreciation was made about it as they are paid for their services.

#### Emotional support

Women at large and more specifically women coming for their first delivery preferred PHCs because they permit a birth companion. This facility has made women to break their custom and deliver in the nearby PHC instead of going to their mother's place for delivery. Most of the public referral hospitals and private hospitals do not permit a birth companion.

“It was my first delivery, I was very scared, my mother wanted to take me to the private hospital, I refused because here (PHC), my aunty will be by my side, and it was a good support.”

“I am scared to go to GH (district hospital), it is a new place, I will be alone in the labour room, with no one by my side—I can't think of that.”

#### Cleanliness of health facility

Clean wards and toilets were another important reason for choosing PHCs. Unclean wards and toilets have been cited as reasons for avoiding the tertiary and secondary centres for birthing care. However, during FGDs, women accepted this due to non-cooperation of the inpatients, who did not keep the toilets clean, ate in the wards, and threw away food wastes in the hospital premises. They felt that this was unavoidable in a hospital where a large number of patients come.

While sharing her delivery experiences in the tertiary hospital, a woman said, “bathrooms and toilets were dirty but we have to go to GH (tertiary centre), it's a matter of life and death, we have to go when PHC refers us.”

FGDs and interviews with mothers revealed that broadly three types of private institutions existed. In one category, individual rooms are provided, good care is given, and the charges are high ranging from INR 12,000 to 15,000 for normal delivery and goes up to INR 40,000 for caesarean sections. Women are happy with such institutions but the cost was beyond their means. The second category charges around INR 6,000 for normal delivery where women share rooms. Here, they said that care is not good, the staff members were not friendly, doctors meet them only once and, above all, the bathrooms and toilets are very dirty. This second category had been chosen many times by the parents of the delivering mother because they think: private facility is good, less expensive and, at the same time, gives an image to in-laws that they have opted for the best care. The third category is the private medical colleges where the hospital is clean, service is good, no delivery charges are needed, and the patient has to pay for medicine and investigations only.

#### Prompt care

Cases coming to the PHCs were immediately attended. Women going to the referral centres had to wait from a few minutes to hours depending on the seriousness of the complication and the availability of staff. Most of the time, they were attended within an hour with a few exceptions as quoted:

“I delivered in the PHC, had severe bleeding, they told me to go to GH (tertiary centre). So, I went there, there were lots of mothers waiting… so, the staff members were not bothered about me. I told them about my problem, they said…. You have already delivered…don't you see there are so many others…. What do you think we can do? Then I went through the back gate, met my husband, and cried…I really suffered, then he took me by an auto to the private hospital.”

#### Confidence in treatment

Many women said that six to seven years back, PHCs referred women to secondary or tertiary-level hospitals for flimsy reasons since ANMs were not confident, and doctors were not available most of the time. Now they felt that the doctors and nurses are more competent and refer them only when required.

“Earlier, even for small problems, they sent us to GH, they will say you are short—go to GH, you are fat—go to GH. Now, it is different, they give good care and send us only if there is a problem and if needed, nurse also comes with us.”

Women had great faith in the treatment provided in the tertiary hospitals. One of them said, “the hospital is not clean, they are not kind to us, they don't start the treatment immediately but they know the right kind of treatment that should be given to us and there is a guarantee for our lives”; this was acknowledged by others also.

Many women feared that doctors in the private hospitals would opt for caesarean section, instead of patiently allowing a woman to go in for normal labour. A few had the same opinion about the tertiary centres also.

One of the reasons for avoiding private sector is the lack of confidence in the doctors. During the interview, a spouse said, “My wife had delivered in the PHC two months back, her first delivery was in a private hospital, there they would write expensive drugs, tonic, and make you pay a lot. Here, they treat with simple drugs. I would suggest any one to come to the PHC only.”

#### Familiarity and proximity to the residence

Women get acquainted with the PHC staff right from their AN visits. PHC staff members take women as a group to the labour room, wards, and toilets and explain to them about the facilities available. Women are convinced about the clean environment and the services available, and this made them to choose PHCs. Proximity to their residence was not the main but an added factor since relatives living nearby can take care of them and their families. Private facilities were also opted due to familiarity with the doctors and staff and proximity to their residence.

### Facilities in the institution

#### Infrastructure and human resources and services

Good buildings, availability of cots, investigation and ultrasonogram (USG) facilities in the PHCs have impressed the public. They said that some six to seven years back, such facilities were not available, staff nurses were not there in all the PHCs, and doctors were not available after 4:00 pm. Now staff nurses are available anytime, and doctors also come whenever needed. The emergency ambulance is used for transport to the public referral centres. However, women opting for private hospitals have to make their own arrangements. Availability of tubectomy services in the block PHCs has made women planning for it to choose PHCs.

Normal deliveries are conducted by nurses in the PHCs and secondary hospitals. In tertiary hospitals, deliveries are conducted by nurses and postgraduate students, under the supervision of the specialists. Obstetricians conduct delivery only in private hospitals where the charges are high. Women are comfortable with nurses conducting deliveries, and they feel that doctors were needed only at times of complications.

All medicines are available in the PHCs; earlier, women were asked to buy iron injections; now it is available in the facility itself. Secondary and tertiary hospitals also provided all medicines; except in rare occasions, women were asked to purchase on their own.

PHCs provide clean linen and provide beds to women till they are discharged. While mentioning about referral hospitals, they said, “We don't always get bed, they put us in the floor on the 2nd or 3rd day, and even if we get the bed they don't change the linen.”

While talking to women about the food provided, they said PHCs provided good food but tertiary hospitals don't provide food. Further probing revealed that tertiary hospitals also provide food that is milk and bread. By some people, only conventional food items for breakfast, like *Idli*, *Pongal*, and rice, were considered as food.

“Four years back, I had my previous delivery in a private hospital, they took good care but I had to buy medicines and pay Rs 5,000 as hospital charges. Now, in the PHC, I get the same type of good care and that too with food and medicine without any cost.”

#### Maternity benefit funds

All women were of a uniform opinion that maternity benefit funds (MBF)—cash incentives given by the Government to promote deliveries in the public facilities—had nothing to do with their choice of facility for delivery. Good quality of care is more important than the cash incentive in choosing a facility as a delivery means life and death to them. Only two out of 16 eligible mothers interviewed had received the state-funded MBF that too, after 3 months.

“The care we get, the cleanliness, facilities in the hospitals, and the kind words of the staff are more important than the MBF. We get the money whenever they give us; of course, we use it for the baby.”

### Cost of care

#### Hospital expenses

Women did not have to spend for medicines or consumables in the public health facilities; Except in a few tertiary hospitals, they were asked to buy some medicines and consumables. Women who delivered in the private hospitals spent from INR 5,000 to 40,000 towards hospital expenses. Most of the hospitals gave bills as a package without break-up details. Women delivering in private and referral hospitals have to spend for their travel and food also.

Women who had earlier delivered in the private hospitals have now moved to the PHCs for subsequent delivery. They said, “When we get the same kind of care in the PHC and that too with food and without expenditure, why should we go to the private hospital?”

#### Informal payment

Women who delivered in the PHCs voluntarily gave around INR 50-100 to the cleaning staff at the time of discharge. No other mode of informal payment was mentioned. Women said that informal payment was rampant in the secondary and tertiary hospitals. Money was paid for better care and help. The total expenses ranged from 1,000 to 2,500, depending on the duration of stay.

“In GH (tertiary hospital), you pay for everything, except doctor's fees, bed charges, and medicines, I paid INR 700 to the lady who brought me to the ward from the delivery room because I had a girl baby; if it were a boy, it would be Rs. 1,000; then for the cleaning staff, stretcher-bearer, and to the gatekeeper everytime when someone comes to see me or bring food.”

## DISCUSSION

The qualitative study design provided insight into the quality of service provision and enabled to get a more holistic understanding of issues surrounding birthing care (9). The opinion shared by women on birthing care was uniform across the three districts, in spite of differences in MMR.

Awareness on the importance of institutional deliveries and availability of services have brought down domiciliary deliveries to near zero. When women have access to more facilities, it is often women's perception of quality care, based on their personal experience, and the opinion they get from their families and friends about the birthing services, which determine their choice of facility for birthing care (10,11). As observed in other studies, spouses and families also have a role in decision-making (11). Complication readiness was lacking, especially saving money as observed in other studies (12). Women and their spouses should be counselled on birth preparedness and complications readiness, to identify danger signs, plan where they should go in times of complication, plan for transport facilities, and also keep some personal savings to prevent delay in seeking care (13).

### Perceptions on quality care

Kind attitude of staff and their willingness to answer to the patient's queries emerged as the key determinant of choice of facility for birthing care. This is where the PHCs score high over the other public facilities. Building a good rapport with the community and making the families gain faith and confidence in the system have been the key strategy for promoting deliveries at PHCs. Important factors identified for client satisfaction with public services were providers’ behaviour, especially respect and politeness (11,14). For women, this mattered more than the technical competence of the providers (14).

Good antenatal care, privacy, cleanliness of the hospital, especially the bathrooms and toilets, and familiarity with the PHC's environment and staff, made women prefer PHCs. Exposing women to the facilities in the labour room and wards in the PHCs during their AN visit—‘maternity picnics’—has paid rich dividends and is a model worth replication by other states to promote deliveries in the PHCs (4,15).

### Infrastructure and human resources

Uncertainty of the availability of staff round-the-clock and their lack of confidence in providing the services leading to frequent referrals have been quoted as major reasons for underutilization of PHCs prior to 2005. The State's initiative of posting three nurses to all the PHCs in a phased manner from 2005 onwards has assured the women on the availability of round-the-clock birthing services (4,7,15). Now, this model has been replicated across the country. The competence of the doctors and the nurses in the PHCs observed by women could be attributed to the in-service training on the management of labour, obstetric first-aid, and newborn resuscitation provided to them (4). Availability of emergency ambulance and the system of nurses accompanying the women to secondary or tertiary hospitals at times of referral have made women more confident to choose PHCs.

From 2005 onwards, the State is in the process of establishing one upgraded PHC in every block—approximately 1 per 100,000 population with five doctors, 30-bedded wards, operation theatres, ultrasonogram (USG), and semi-auto analyzers to provide for all investigations. Currently, 309 out of 385 blocks have one upgraded PHC with the above facilities. In addition to the upgraded PHCs, another 700 PHCs are also provided with semi-auto analyzer and USGs. Construction of new PHCs and repairs in the existing PHCs have also been taken up. Equipment for newborn care, basic investigations, and adequate drugs are available in all the PHCs (8). All PHCs conduct normal deliveries. A few PHCs have started performing elective caesarean sections (7). However, it has to be mentioned that, prior to 2005, the PHCs also had the basic facilities to conduct normal deliveries and, yet, it was underutilized (4,16).

### Accountability of service providers

Same doctors and nurses who work in the PHCs later move to the secondary and tertiary hospitals; yet, vast difference is observed in attitude and behaviour of staff. What made PHC staff more courteous and accountable bringing a four-fold increase in deliveries in the PHCs is the question before us.

This could be because maternal and child health is the main focus of PHCs and the availability on an enabling environment in the PHCs. State-level monthly review of district officers focuses on delivery performance of PHCs, and districts are ranked based on that, which makes PHC staff more accountable (17). This monitoring mechanism may be a catalyst to creating a women-friendly environment in the PHCs. Once such systems are established, the demand from the community sustains this. Better-performing district administrators and PHC providers get due recognition from the public, politicians, and bureaucrats (4). Such accountability is lacking in the referral centres since delivery is one of the many services provided there.

Proximity to the residence is another factor favouring choice of PHCs as seen in other studies; yet, it is not a key factor (1,9,10) since the State has a good transport network system and women can reach the secondary and tertiary hospitals in most places within 30 to 40 minutes and can have access to specialist care. In Tamilnadu, women's choice of a facility for birthing is based on a user-friendly environment and faith in the services provided. They have confidence in the nurses who conduct deliveries, unlike Kerala, where most of the deliveries are conducted in the presence of doctors. In Kerala, 63% of the deliveries are conducted in the private sector. In the public sector, most of the deliveries take place in the secondary and tertiary hospitals, and not many in the PHCs (18,19). This is contradictory to scenario in Tamilnadu where 67% of the deliveries take place in the public sector, and PHCs alone contribute to 27.2% (8,19).

### Maternity benefit funds

Women from below poverty-line delivering in all public health facilities are eligible for cash incentive of INR 12,000 under state-funded Dr. Muthulakshmi Reddy Maternity Benefit Fund in addition to JSY. Cash incentives have prompted in seeking delivery care at health facilities but an enabling environment and courteous behaviour of providers score over the cash incentives. A study on women's perceptions of the quality of maternal care in Jharkhand showed that perception of better care and outcomes override the attraction of cash transfer in women's preference for institutional deliveries (11). Registration of women for MBF can be considered only an opportunity to promote deliveries in the PHCs. Women whose families felt that private sector provides good care still opt to go there, willing to forego the MBF. States should focus on strategies for creating an enabling environment for the women to use the facilities and educate women to demand for services rather than relying more on cash incentives to ensure sustainability.

### Other facilities

In view of the benefits of having a birth companion, Government of Tamilnadu has passed an order to permit a birth companion in all public hospitals (5). Unlike most of the referral hospitals, all PHCs adhere to this and is one of the reasons which made women delivering for the first time to opt for PHCs, instead of the private sector. Provision of food, minimal informal payment, and availability of family planning services in the PHCs has attracted women having their second or third delivery to opt for PHC (4).

Many studies and reports have highlighted the strengths of the PHCs but are not free from system constraints, like understaffing, non-stayal of doctors, and gaps in other managerial issues exist (4,15,19). In one study, the authors have mentioned that toilet was not clean, and labour room was crowded in the PHC they visited (15). Another report has also mentioned about the water scarcity in a PHC (19). Inadequacies in the PHCs were not mentioned by women probably because such problem existed in a very few centres, and this study focused on birthing care only. However, the scope for further studies of health systems challenges is evident (20).

### Barriers to seeking care from referral hospitals and private sector

Barriers to seeking care in the secondary and tertiary public hospitals and private hospitals have made PHCs the preferred option for birthing care in the State. The secondary and tertiary-level hospitals have gained the confidence of the public as far as treatment is concerned. However, issues, like unkind attitude of staff, lack of privacy, non-availability of birth companion, unclean hospital premises and toilets, and high informal payment in most of the hospitals, have been quoted as a dissuader for women in choosing the facility for subsequent delivery. Similar views have been reflected in other studies also (20–22); such women's experiences are shared with friends and relatives and create a fear among others to choose the facility for delivery as seen in other studies. In spite of such barriers, studies have shown that the public services in the State are rated higher than the private sector (23).

Fear of unnecessary caesarean sections, high expenses, and non-availability of a birth companion were reasons for reluctance in opting for private sector. High incidence of caesarean section rates is observed in the State, especially in the private sector (19).

### Challenges

In spite of providing good care and satisfying the user's needs, there are a few challenges which need to be addressed. One of the major challenges is the weak linkage between the PHCs and the referral hospitals. There is poor feedback to the PHCs from the referral centres. Referral linkages should be strengthened and periodic meetings arranged to develop acquaintances between the PHC and referral hospital staff. Doctors in the referral hospitals should provide mentoring support to the PHC staff.

There is a continuous transfer of doctors and staff nurses from the primary to the secondary and tertiary hospitals; the new recruits should be empowered with adequate skills to sustain optimal utilization of PHCs.

In view of the facilities in the PHCs, Village Health Nurses promote deliveries at PHCs as observed in other studies (9), resulting in a shift of deliveries from HSCs to the PHCs. Effective utilization of primary-level public institutions for normal deliveries is always recommended as it is convenient for the women to get the service close to their residence; besides, it helps bring down domiciliary deliveries and reduce the out-of-pocket expenses. Good healthcare systems should promote increased utilization of PHCs as it leads to reduced case-load of normal deliveries in the secondary- and tertiary-care centres; this would help the referral centres to focus on high-risk cases demanding more facilities (24,25).

### Conclusions

The study has highlighted that courteous behaviour and good interpersonal communication of staff were the key determinants of choosing PHCs for birthing care. Good infrastructure, clean and familiar environment, confidence in the service provided, and less informal payment are added factors favouring choice of PHCs. Provision of such women-friendly services free of charge, with easy access, has made PHCs the preferred option for birthing care. The barriers to seeking care from public referral hospitals and private hospitals have also made women to prefer PHC. New staff should be empowered with adequate skills to sustain adequate utilization of PHCs. It is indispensable to establish a good referral linkage as women need to use the PHCs for normal delivery and have to move to the secondary and tertiary level in case of complications to reduce maternal mortality and morbidity. The findings would be a guide to the decision-makers to take into account women's perception of care while planning for services leading to better utilisation of services and improved outcomes.

**Conflict of inetrest:** Authors declare no conflicts of interest.
